# Identifying genetic markers for a range of phylogenetic utility–From species to family level

**DOI:** 10.1371/journal.pone.0218995

**Published:** 2019-08-01

**Authors:** Bokyung Choi, Michael D. Crisp, Lyn G. Cook, Karen Meusemann, Robert D. Edwards, Alicia Toon, Carsten Külheim

**Affiliations:** 1 Division of Ecology and Evolution, Research School of Biology, The Australian National University, Canberra, Australian Capital Territory, Australia; 2 The University of Queensland, School of Biological Sciences, Brisbane, Queensland, Australia; 3 Evolutionary Biology & Ecology, Institute for Biology, University of Freiburg, Freiburg (Brsg.), Germany; 4 Australian National Inset Collection, CSIRO National Research Collections Australia, Canberra, Australian Capital Territory, Australia; 5 Department of Botany, National Museum of Natural History, Smithsonian Institution, Washington, D.C., United States of America; 6 School of Forest Resources and Environmental Science, Michigan Technological University, Houghton, Michigan, United States of America; East Carolina University, UNITED STATES

## Abstract

Resolving the phylogenetic relationships of closely related species using a small set of loci is challenging as sufficient information may not be captured from a limited sample of the genome. Relying on few loci can also be problematic when conflict between gene-trees arises from incomplete lineage sorting and/or ongoing hybridization, problems especially likely in recently diverged lineages. Here, we developed a method using limited genomic resources that allows identification of many low copy candidate loci from across the nuclear and chloroplast genomes, design probes for target capture and sequence the captured loci. To validate our method we present data from *Eucalyptus* and *Melaleuca*, two large and phylogenetically problematic genera within the Myrtaceae family. With one annotated genome, one transcriptome and two whole-genome shotgun sequences of one *Eucalyptus* and four *Melaleuca* species, respectively, we identified 212 loci representing 263 kbp for targeted sequence capture and sequencing. Of these, 209 were successfully tested from 47 samples across five related genera of Myrtaceae. The average percentage of reads mapped back to the reference was 57.6% with coverage of more than 20 reads per position across 83.5% of the data. The methods developed here should be applicable across a large range of taxa across all kingdoms. The core methods are very flexible, providing a platform for various genomic resource availabilities and are useful from shallow to deep phylogenies.

## Introduction

Over the past couple of decades, as molecular techniques and resources have evolved, plant phylogeneticists have begun to employ greater numbers of nuclear and chloroplast loci, often in concert, to estimate species relationships. Chloroplast loci have been widely used due to their simple and stable structure and ease of primer design and amplification [1]. The uniparental inheritance of chloroplast loci has the disadvantage that they are not necessarily tracking the species tree and their evolutionary rate is more conserved [1–4]. Nuclear genomes provide many independent and unlinked loci that evolve at different rates. They generally evolve faster than chloroplast loci [5, 6], but have a disadvantage due to lack of available genomic data for most taxa. Further complications arise from the presence of gene duplications or gene loss, such that paralogy is often found when a nuclear locus is sequenced in another taxon [7]. Furthermore, the slow rate of DNA evolution in cpDNA and most protein-coding nuclear loci means that having access to only a few loci does often not resolve species-level phylogenies. Consequently, many plant phylogenies are based on multiple cpDNA loci with only the ITS regions representing the bi-parentally inherited nuclear loci. To obtain well resolved phylogenies for species-rich plant genera requires identification and sequencing of many nuclear loci, which in combination with cpDNA can be advantageous, making use of varied rates of evolution as well as potentially different evolutionary histories [8]. When targeting loci for highly diverged taxa we expect exons to be more reasonable candidates compared to intronic or noncoding regions because they have less variation and can be aligned more reliably than intronic and non-coding regions that may have excessive length variations and higher substitution rates [9].

However selecting the best loci for such work remains difficult and finding sets of loci that inform at different levels of evolutionary history involves resolving a tradeoff between informativeness and alignability. It is also important that loci are orthologous, i.e. shared due to common ancestry and not paralogs from duplication events [7, 10]. Single or low copy-number nuclear genes are thus desirable as the chance for the occurrence of paralogs is much reduced [11].

A number of different approaches have been taken to extract suitable loci, mostly in the form of reduced representation methods [12]. These often use restriction enzymes to target a subset of the genome and include Reduced Representation Library (RRL), Restriction-site Associated DNA Sequencing (RAD), and Genotyping-By-Sequencing (GBS), and have the advantage of not requiring genomic reference data, but typically do not work well for distantly related taxa because restriction sites tend to be less conserved [13].

More specific methods of reduced representation involve target enrichment including PCR-generated probes [14], developing markers that mainly target 3’UTR and coding regions [15], or transcriptome-based exon capture [16]. Even more targeted methods involve using existing genomic resources to identify candidate genes—Bragg et al. [12] used *Anolis* lizard exons to identify homologs in transcriptomes from more distantly related taxa using reciprocal best BLAST, while Li *et al*. [17] took a genome comparison approach comparing exons across fish genomes to filter putatively single-copy orthologous exons. These methods may be useful for phylogenetic estimation across more diverged taxa [12, 18].

Similarly, targeting Ultra-Conserved Elements (UCEs) via hybrid enrichment can be useful for resolving intermediate to deep level phylogenies [19], however these do not always provide enough signal to resolve shallower nodes (e.g. [20]). Marker sets used for anchored phylogenomics (a variation of the hybrid enrichment approach [21]) can be useful across multiple phylogenetic levels as both conserved loci and flanking regions containing variation can be targeted, but has a relatively long development time [19]. Likewise, exon capture has been commonly used for phylogenetics at multiple levels [19] but requires prior genomic resources such as a reference genome(s). Identifying numbers of informative loci with limited pre-existing genomic resources remains a challenge. Recent method developments to find conserved loci for phylogenetic inferences in plants include hybrid methods based on both transcriptome and genome skim data in Oxalidaceae [22, 23], or transcriptome and whole genome sequence data in Ericaceae [24].

The Myrtaceae is a large family of trees and shrubs (~6,000 species in over 130 genera) distributed throughout tropical and warm-temperate regions worldwide. Thirteen tribes with a range from 1 species (Lindsayomyrteae) to 3279 species (Myrteae) are recognized [25]. Although the crown age of the family is estimated at 75–93.5 Ma, much of the diversification has been within the last 20 million years and is centered in Australia with two genera, *Melaleuca* and *Eucalyptus*, representing over 1,000 species and with an estimated divergence of 63–72.8 Ma [26]. They form an important element of the flora and are ecologically and economically valuable. To date phylogenies for *Eucalyptus* and *Melaleuca* have been estimated only from small numbers of nuclear and/or chloroplast loci [26–34] with limited success in resolving relationships at the species level. Recent radiation of many species groups resulting in incomplete lineage sorting and/or ongoing hybridization have been identified as likely causes for a lack of resolution in *Melaleuca* [29], *Corymbia* [35] and *Eucalyptus* [27] with similar issues expected across the family. Myrtaceae thus present a challenge for identifying loci that are low copy and informative for robust phylogeny reconstruction through the depth of the tree.

The method we present here is a target capture approach aimed at identifying numbers of orthologous low-copy loci from both the nuclear and chloroplast genomes that are potentially useful for resolving species level relationships across a large family (Myrtaceae), with the expectation that it can be extended to many other groups.

## Material and methods

### *Melaleuca* RNA sequencing and read processing

**Fig 1:**
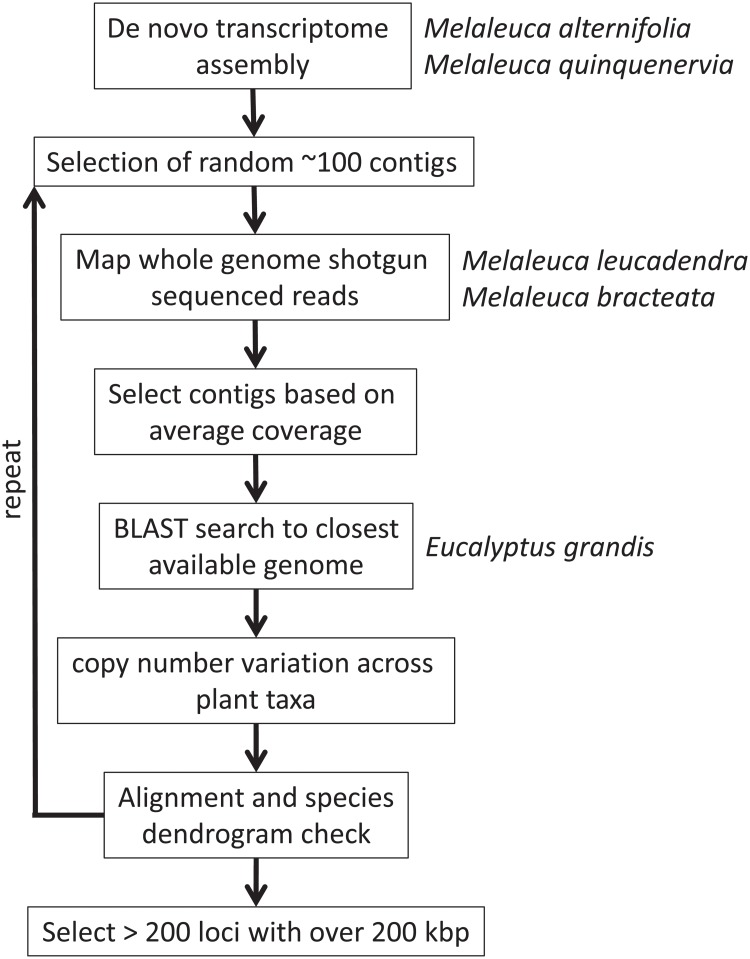
Flow chart of the locus discovery pipeline.

An assembled transcriptome sequence from leaves of *Melaleuca quinquenervia* was provided by Sarah Hsieh [36]. In brief, RNA was extracted from leaf tissue of each of 16 individuals (8 per species) at two different time points in a plant-fungal interaction experiment. Libraries were prepared from total RNA using the TruSeq RNA sample preparation kit v2 (Illumina, San Diego, USA) and sequenced on the Illumina HiSeq2500 platform with a 150 bp paired-end read protocol. After assessing the read quality, and trimming of adaptors and low quality reads, Trinity was used to assemble the transcriptome *de novo* [37] for each species followed by selection of the longest isoform using CD-EST-HIT [38] as described in [36]. We selected about one hundred random contigs from these assembled transcriptomes at a time for the steps outlined below (Fig 1). For further comparison, we also used an assembled transcriptome from *M*. *alternifolia* provided by Sarah Hsieh, which was prepared, sequenced and analysed in the same way as *M*. *quinquenervia* (Hsieh et al. unpublished).

### Whole-genome shot-gun sequencing of two distantly related individuals of *Melaleuca*

To ensure target loci are present in low or single copy number in other species of *Melaleuca*, we shot-gun sequenced the genomes of two species of *Melaleuca*: *M*. *leucadendra*, which is closely related to *M*. *quinquenervia*, and *M*. *bracteata*, which is distantly related to both *M*. *quinquenervia* and *M*. *alternifolia* [31]. Genomic DNA was extracted from *M*. *bracteata* and *M*. *leucadendra* (S1 Table) using a CTAB extraction protocol (modified from [39] combined with a Qiagen spin column protocol (DNeasy plant mini kit, Qiagen) to maximize the quantity and quality of DNA. The extracted DNA was sent to Macrogen (Republic of Korea) for library preparation and sequencing. The library was prepared using a TruSeq DNA LT Sample Preparation Kit (Illumina, San Diego, USA) following the manufacturer’s instructions. The samples were sequenced using the Illumina HiSeq2500 platform with a 150 bp paired-end read protocol. We received 42,756,290 reads for *M*. *bracteata* and 44,538,892 reads for *M*. *leucadendra* resulting in a genome coverage of ~16 X for *M*. *bracteata* and ~18–22 X for *M*. *leucadendra*. These estimates were based on both mapping reads against the contigs as described below and a Myrtaceae genome review by [40].

### Identification of target genetic loci

Low quality bases and reads were removed from the raw reads in CLC genomics workbench v 4.6 (CLC bio, Aarhus, Denmark) using the standard parameters for low quality base removal (limit of 0.05, maximum 2 ambiguous nucleotides) and Illumina adapter trimming. We mapped the whole genome shot-gun sequences from each of *M*. *leucadendra* and *M*. *bracteata* to the ca. one hundred RNAseq contigs for each iteration using CLC genomics workbench v 4.6 with standard parameters (CLC bio, Aarhus, Denmark). These 100 contigs were retrieved from the *de novo M*. *quinquenervia* transcriptome assembled as described above. The median read-mapping depth of *M*. *leucadendra* and *M*. *bracteata* was 18.4 and 15.7 reads per nucleotide position, respectively. Each of the 100 contigs was then filtered based on being within one stdev of the mean coverage for both species. This step was meant to exclude loci that were either not present or partially absent in the genomes of *M*. *leucadendra* and *M*. *bracteata* (using the criterion of coverage well below the mean), or if the contig belonged to a gene family or contained repetitive sequences (coverage well above the mean due to reads belonging to other genomic regions mapping to the target locus). Every contig that matched the above criteria was then BLASTed against the genome sequence of *Eucalyptus grandis* (using BLASTN) in Phytozome V10 (www.phytozome.com) and was kept if exactly one similar gene (best hit E value < 1e^-50^, second lowest E value > 0.1) was found. This putative ortholog from *E*. *grandis* was then compared to 68 other sequenced and annotated green plant genomes [41] using the gene ancestry function in Phytozome V10. This allowed the further exclusion of loci that occur in some plant species as medium to large gene families. Loci with a range of 1–6 copies per genome across the 68 species were selected. Sequences of putative orthologous loci from *E*. *grandis*, *M*. *leucadendra*, *M*. *bracteata*, *M*. *alternifolia* and *M*. *quinquenervia* were then aligned using Geneious alignment with standard parameters in Geneious v7.1.4 (http://www.geneious.com, [42]). We visually assessed each alignment to discard candidate loci if less than 30% of the sequence length was aligned. A species dendrogram was built for each locus using a Tamura-Nei Genetic Distance Model and the Neighbor-Joining method in Geneious (v7.1.4). Potential paralogs were filtered out by excluding loci where the gene dendrogram did not show the known phylogenetic species relationships in Edwards *et al*. [31] and Thornhill *et al*. [26]. Loci were excluded if *E*. *grandis* was not sister to the *Melaleuca* species [26], or if *M*. *leucadendra* and *M*. *quinquenervia* were not sister species [31]. Iterations of this procedure were repeated from randomly selected *M*. *quinquenervia* contigs until over 240 kbp of nuclear loci were selected (179 loci). We tested this pipeline based on *M*. *alternifolia* contigs and found similar results, though contigs selected through this species were not maintained for the downstream probe design.

The chloroplast genome sequence of *E*. *grandis* (http://www.ncbi.nlm.nih.gov/genbank/) and shot-gun sequences of *M*. *leucadendra* and *M*. *bracteata* were aligned using the Mauve alignment using standard parameters in Geneious v7.1.4 (http://www.geneious.com, [42]). After manual adjustment of the chloroplast alignment, we selected regions that contained at least nine single nucleotide polymorphisms for each 500 bp segment (29 regions selected). We also included the *trnL-trnF* region, the *psbA-trnH* intergenic space region, the *matK-trnK* region and the *ndhF* gene that have been used in previous phylogenetic studies of Myrtaceae.

### Probe design

Both the chloroplast and nuclear probes were designed by NimbleGen (Madison, USA) using their standard parameters of the SeqCap EZ System based on both an *E*. *grandis* set of loci and the same set from *Melaleuca* consensus sequences, so that every locus was represented twice in the design. Probe design was adjusted for nuclear or plastid origin of loci by reducing the plastid tiling density. The probe length, which was determined by sequence context, ranged approximately from 55–105 mers.

### DNA extraction and library preparation

Genomic DNA (gDNA) was extracted from leaf tissues of 48 samples from five genera and 43 species of Myrtaceae (Table 1) using the Qiagen DNeasy 96 Plant Kit (Qiagen, Hilden, Germany). Represented were: *Arillastrum* (one species), *Corymbia* (four species), *Eucalyptus* (31 species), *Heteropyxis* (one species) and *Melaleuca* (five species). We also included a *Calothamnus* species because our previous work showed all genera in the tribe Melaleuceae to be included in *Melaleuca* [31, 43]. The gDNA was quantified using either the LabChip DS Spectrophotometer (Trinean, Gentbrugge, Belgium) or by separating them by agarose gel electrophoresis. The DNA for each sample was diluted to 600 ng in 100 μl. The diluted gDNA was sheared for a target size ranging between 250–400 bp using the Bioruptor ultrasonicator (Diagenode, Liege, Belgium) with the following settings: 10 cycles of 30 s on/off at high intensity (S1 Protocol).

**Table 1:** Number of reads, percentage of reads mapped back to the *Eucalyputus grandis* targets and the average coverage of 47 samples (chloroplast, nuclear and total). P-distance (p-dist) of each species compared to *Eucalyputus globulus* subsp. *pseudoglobulus*.

Collector no	Species	# reads	Mapped to target (%)	cov. (cpl)	cov. (ncl)	cov. (total)	P-dist
McCoy1995a	*Arillastrum gummiferum*	1594438	57.2	230	220	221	0.1
McCoy1995b	*Arillastrum gummiferum*	849038	60.2	105	126	123	0.1
Cs229	*Corymbia bunites*	2785808	56.9	389	383	384	0.11
Cs377	*Corymbia calophylla*	18232	58.1	8	2	3	0.47
Cs380	*Corymbia haematoxylon*	416744	59.7	187	49	71	0.12
Cs493	*Corymbia peltata*	2237606	55.5	545	277	318	0.11
Cs474	*Eucalyptus brockwayi*	1213666	62.1	326	180	203	0.06
ANBG9404806	*Eucalyptus sp*.	1250566	55.2	235	169	180	0.04
CANB632680	*Eucalyptus extrica*	1028128	64.9	451	142	191	0.06
Cs729	*Eucalyptus globoidea*	679956	61.8	52	109	100	0.06
CANB688145	*Eucalyptus globulus*	1005544	58.1	145	144	144	0.04
CANB494954	*Eucalyptus globulus*	307928	52.8	18	41	37	0
Cs357	*Eucalyptus gomphocephala*	1085564	58	189	153	159	0.05
Cs424	*Eucalyptus goniantha*	17766	60.6	3	3	3	0.45
Cs496	*Eucalyptus howittiana*	715782	58.8	186	100	114	0.05
Cs275	*Eucalyptus insularis*	586980	60.1	100	86	88	0.06
CANB413807	*Eucalyptus insularis*	351106	65.8	25	59	54	0.06
Cs716	*Eucalyptus leucoxylon*	862498	658.2	163	123	130	0.05
Cs721	*Eucalyptus ligulata*	100490	61.5	20	15	16	0.1
Cs574	*Eucalyptus moorei*	1360608	58.5	221	192	196	0.06
Cs744	*Eucalyptus nitada*	603824	55.4	109	79	84	0.06
Cs708	*Eucalyptus optima*	285804	60.3	52	42	44	0.05
Cs305	*Eucalyptus pachycalyx*	52466	59	17	7	9	0.14
Cs426	*Eucalyptus pachyloma*	398460	63	84	60	64	0.06
Cs742	*Eucalyptus perriniana*	369972	60.3	120	53	64	0.04
Cs482	*Eucalyptus pilularis*	1413500	62	262	205	214	0.06
CANB638520	*Eucalyptus platydisca*	1465552	62	229	221	222	0.06
Cs431	*Eucalyptus pleurocarpa*	667054	62.2	176	96	109	0.06
Cs425	*Eucalyptus preissiana*	121636	58.7	34	17	20	0.08
Cs227	*Eucalyptus pumila*	1299962	60.9	219	197	201	0.05
CANB632673	*Eucalyptus selachiana*	344242	64.8	144	47	62	0.07
CANB693174	*Eucalyptus selachiana*	1070662	59.5	172	154	157	0.07
Cs368	*Eucalyptus spathulata*	1080026	56	133	151	148	0.05
Cs366	*Eucalyptus staeri*	209512	60.2	19	32	30	0.07
Cs733	*Eucalyptus stenostoma*	31016	59.9	5	5	5	0.29
Cs191	*Eucalyptus stoatei*	1225546	59.3	262	177	190	0.05
Cs769	*Eucalyptus tereticornis*	1691082	60.4	105	269	243	0.05
Cs715	*Eucalyptus verrucata*	438384	60.7	98	63	68	0.06
Cs727	*Eucalyptus sp*.	313464	66.7	33	54	50	0.06
Cs492	*Eucalyptus willisii*	369260	63.2	122	52	63	0.06
CANB576168	*Heteropyxis natalensis*	149702	31.9	60	8	16	0.43
RDE176	*Melaleuca capitata*	3455370	48.9	1210	357	492	0.14
Harwood1546	*Melaleuca cornucopiae*	4366802	44.3	711	449	490	0.14
AF4308	*Melaleuca foliolosa*	1434504	48.1	288	159	179	0.15
RDE75	*Melaleuca squarrosa*	1971326	47.8	308	228	240	0.14
AF4286	*Melaleuca sylvana*	1671790	47.3	212	194	197	0.14
RDE154	*Calothamnus gracilis*	1634982	41.4	469	139	191	0.14
	**AVERAGE**	991582	57.6	197	130	140	0.11

We used the Rohland and Reich [44] protocol with minor modifications in reagents and incubation settings for sequencing library preparation (S1 Protocol). After the enrichment PCR, three samples were randomly selected for Qubit fluorometric quantification using the dsDNA HS Assay kit (Qubit Invitrogen, Carlsbad, USA). All 48 samples were quantified by agarose gel electrophoresis. We pooled between 7–11 μl from each sample depending on the quantification to obtain a total of ca. 1.25 μg DNA (S1 Protocol).

### Target hybridization, recovery, wash, and sequencing

We hybridized the pooled DNA library of 48 samples to the target probes using the SeqCap EZ Developer Library (NimbleGen, Madison, USA) following the manufacturer’s instructions with minor modifications in the hybridization mix preparation and incubation settings (S1 Protocol). The main modification was to denature the DNA library and hybridization mix for 10 minutes at 95°C, then gradually decrease the temperature from 95°C to 47°C followed by a 47°C incubation for 72 hours. This was to allow the formation of uniform ssDNA of both probes and library. Recovery and wash of hybridized samples was carried out using the SeqCap Hybridization and Wash Kit (NimbleGen, Mannheim, Germany) following the manufacturer’s instructions with a slight modification in temperature settings (S1 Protocol).

We performed semi-qPCR to quantify the DNA in the hybridized library and estimate the number of cycles for the indexing PCR step. After the indexing PCR of captured libraries, samples were purified with Sera-mag SPRI beads to remove primer dimers from the indexing PCR product. We quantified the concentration of the hybridized libraries using the Qubit fluorometer (Qubit Invitrogen, Carlsbad, USA). The captured library was then sequenced on the Illumina Miseq platform (100 bp paired-end read protocol) at the Bio-molecular Research Facilities at The Australian National University.

### Data handling and mapping of reads

**Fig 2:**
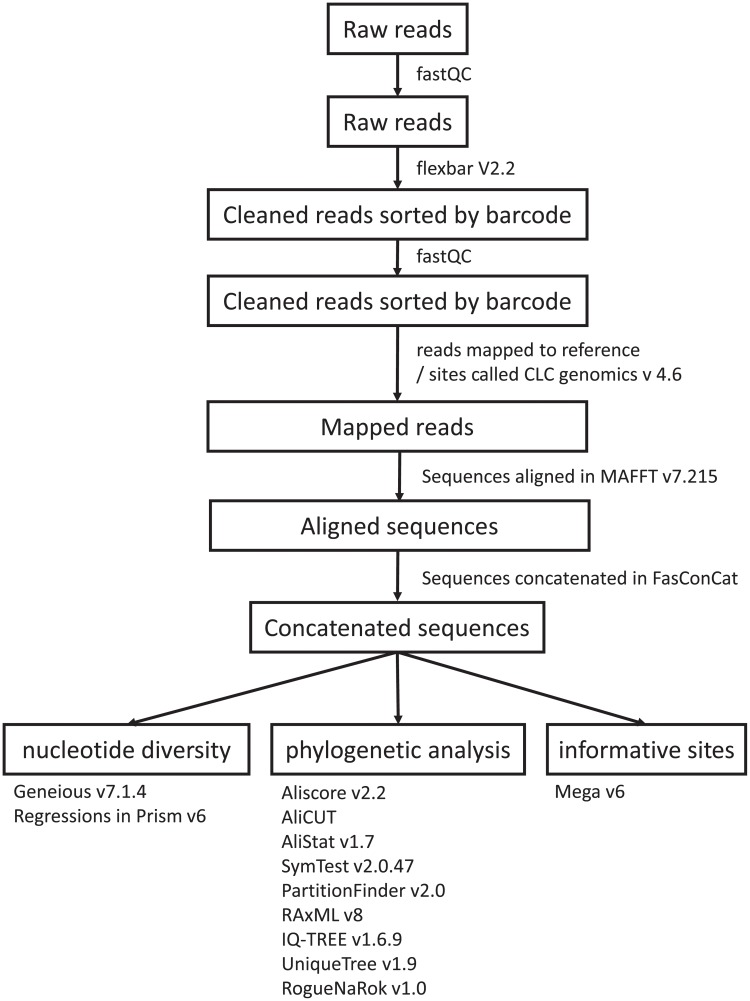
Flow chart of the bioinformatics analysis pipeline.

Fig 2 shows the data analysis pipeline. The quality of the raw reads was investigated using FastQC (http://www.bioinformatics.babraham.ac.uk/projects/fastqc/). We used Flexbar V2.2 [45] to sort reads by barcodes, and to remove barcodes and low quality reads using standard parameters and a text file containing the 48 unique barcodes for each sample. The processed reads were double-checked using FastQC for the quality of the cleaned reads. The reads were mapped against the *E*. *grandis* targets using the CLC genomics workbench v 4.6 with standard mapping parameters (CLC bio, Aarhus, Denmark). We obtained data for 209 of the 212 nuclear and chloroplast loci. We called sites in CLC genomics by simple majority rule with a minimum of 60% of reads required to be one allele. One of the three *Arillastrum* samples was excluded from the subsequent analyses due to a potential sample mix-up or mislabeled garden specimen. It did not cluster with the vouchered two specimens collected from a natural population in New Caledonia.

### Nucleotide diversity estimation

After renaming the sequence headers to the respective sample name, we aligned sequences of the 47 taxa for each locus using MAFFT L-INSI [46] in MAFFT v.7.215 (http://mafft.cbrc.jp/alignment/software/). Then the alignments of 209 loci were concatenated using FasConCat (https://www.zfmk.de/en/research/research-centres-and-groups/fasconcat). The nucleotide diversity across the 47 samples of 209 loci compared with *Eucalyptus globulus* subsp *pseudoglobulus* was calculated using Geneious v7.1.4 (http://www.geneious.com, [42]). Nucleotide diversity was compared to the percentage of reads that mapped back to the *E*. *grandis* reference using linear regression in Prism version 6 (GraphPad). We compared the GC content of each locus (calculated in Geneious v7.1.4) with the average per locus coverage to test whether GC content affected target capture.

The number of parsimony-informative sites (= informative sites) for each locus was calculated in Mega v6 [47]. We assessed both the number of informative sites and the percentage of informative sites per target length. The number of informative sites is not necessarily a definite measure of phylogenetic utility as this would depend on which phylogenetic analyses are selected. Nevertheless, the number of informative sites can be used as an approximate measure of phylogenetic usefulness for each locus (e.g. [18]).

### Phylogenetic analysis and maximum likelihood tree inference

Multiple sequence alignments were evaluated against randomly similar aligned sequence regions with Aliscore [48, 49], version 2.2, with the default parameters: sliding window size and number of sequence pairs set to the maximum (option–r). Alignment sections identified as randomly similar or ambiguously aligned were excluded with AliCUT (available from https://github.com/PatrickKueck/AliCUT). A small percentage (2.3%) was discarded. Masked multiple sequence alignments were concatenated into a supermatrix with FasConCat version 1.0 [49] spanning an alignment length of 257,799 sites.

The supermatrix was further explored using AliStat v. 1.7 (available from: https://github.com/thomaskf/AliStat) with respect to the coverage of the entire dataset and considering pairwise sequence comparisons. Coverage by this measure was also very high (Completeness (C) score for the alignment (Ca): 94.3%; max. C-score for individual sequences (Cr_max): 98.9%, min. C-score for individual sequences (Cr_min): 55.3%).

To explore whether or not the dataset matched stationary, homogeneous and time reversible (SRH) conditions (e.g. [50, 51]), we applied the Bowker’s matched-pairs test of symmetry as implemented in SymTest version 2.0.47 (available from: https://github.com/ottmi/symtest) considering all three codon positions. SRH conditions matched for most of the sequence pairs except for few *Eucalyptus* and *Melaleuca* sequence comparisons.

We subsequently applied the software PartitionFinder version 2.0 [52], pre-release 2.2, using the implemented RAxML version 8 [53] and testing the substitution models GTR and GTR+G with empirical base frequencies for each of the 209 loci, in order to merge single loci partitions into meta-partitions due to an improved AICc score [54]. We applied the “rcluster” algorithm with linked branch lengths. The best partition scheme revealed 134 meta-partitions. Before inferring maximum likelihood trees, we re-estimated the best fitting of all available substitution models and nucleotide models for each meta-partition (i.e. the best partition scheme) with Modelfinder [55] as implemented in IQ-TREE (v.1.6.9), with linked branch lengths, choosing the AICc for model selection and considering the edge-proportional partition model allowing meta-partitions to have different evolutionary rates (option -ssp).

For maximum likelihood (ML) tree inference, we performed 50 independent tree searches (25 with a random start tree and 25 with a parsimony start tree) with IQ-Tree v 1.6.9 [56, 57]. We used the best partition scheme and respective models selected in the previous step. All ML trees showed one unique tree topology (assessed with Unique Tree v.1.9, kindly provided by T. Wong and available upon request). We calculated branch support via non-parametric bootstrapping (100 bootstrap replicates with random start trees and the option -ssp, see above) and mapped bootstrap support onto the ML tree with the best log-likelihood. We ensured bootstrap convergence as described in [58] a posteriori with RAxML (v.8.2.11) ([53] settings:”autoMRE”, -B 0.03,—bootstop-perms = 10,000, performing the test ten times with different random seeds). Bootstrap convergence was always fulfilled after 100 bootstrap replicates.

We additionally tested for the occurrence of rogue taxa with RogueNaRok (v.1.0) [59] using the best ML tree. We identified our dataset as free from any rogue taxa. The best ML tree was rooted with *Heteropyxis natalensis* (from subfamily Psiloxyloideae) using SeaView (v.4.5.4) [60]. The best tree was graphically edited with Inkscape (v.0.91) (www.inkscape.org) and Illustrator-EPS with bootstraps mapped on branches.

## Results

We successfully identified loci for target capture in the plant family Myrtaceae and captured and sequenced the target loci from 47 samples representing 43 species across five genera (*Melaleuca s*.*l*., *Eucalyptus*, *Corymbia*, *Arillastrum*, *Heteropyxis*) (Table 1). We recovered 209 nuclear and chloroplast loci consisting of 263,164 bp from the 212 target loci (S1 Table). We sequenced 176 nuclear loci with a combined length of 241,047 bp, 0.39% of the *E*. *grandis* nuclear genome, and 33 chloroplast loci with a combined length of 22,117 bp covering 13.8% of the *E*. *grandis* chloroplast genome. The length of chloroplast and nuclear target loci ranged from 216 bp to 6,555 bp with the majority between 500 to 2,000 bp in size (S2 Table).

### Performance of exon capture

**Fig 3:**
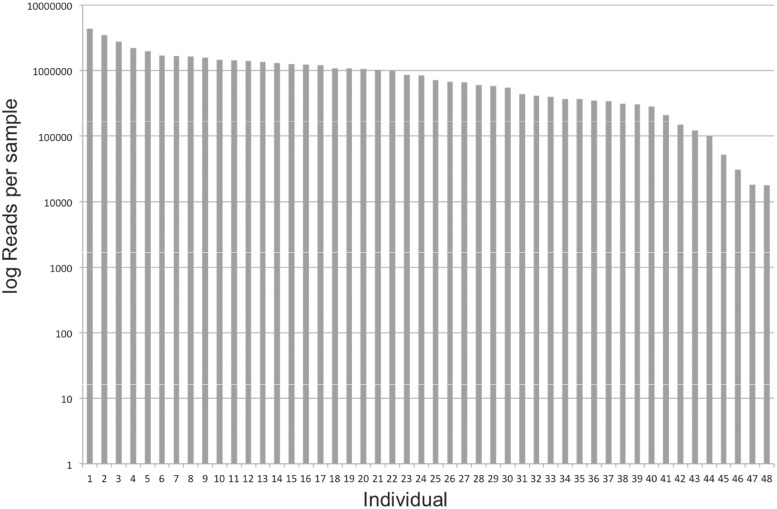
Number of reads per sample mapped back to the *Eucalyptus grandis* targets, sorted from high to low.

The total number of reads for the 209 loci across 47 samples was 47,707,942. The number of reads filtered by barcodes that are unique to each sample ranged from 17,766–4,366,802, mean of 991,582 (Table 1, Fig 3). There were 387,400 reads without a valid barcode. The following three criteria were used to assess the target capture performance and sequencing: 1) capture specificity, 2) capture sensitivity and 3) enrichment factor (EF).

Capture specificity refers to the percentage of reads that mapped back to the target sequences [14]. The percentages of reads that mapped back to the *E*. *grandis* targets ranged from 31.9–66.7% for the different samples, with an average of 57.6% (Table 1). The average capture specificity for each genus was: *Eucalyptus* (60.2%), *Arillastrum* (58.7%), *Corymbia* (57.6%), *Melaleuca* (46.3%), and *Heteropyxis* (31.9%).

**Fig 4:**
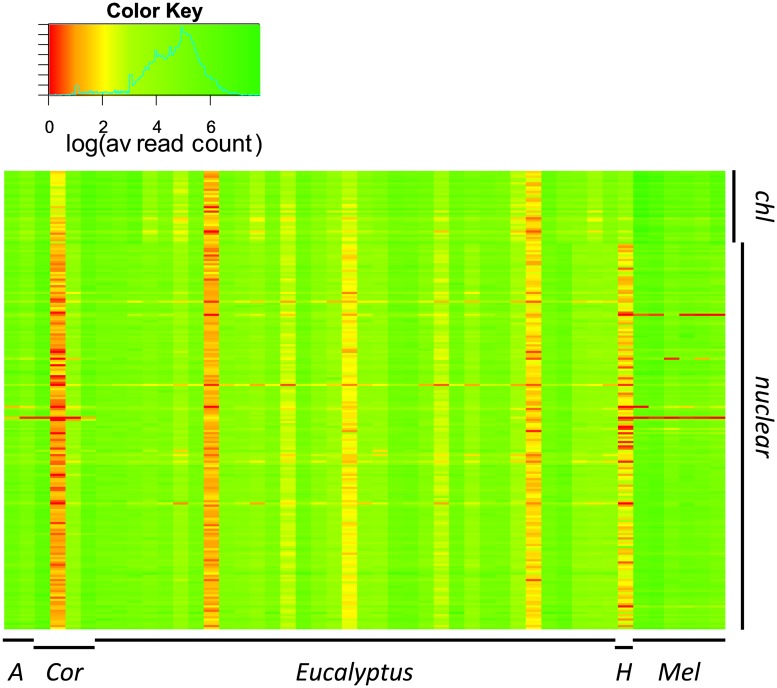
A heat map of the average coverage across 209 loci and 47 samples. Average reads mapped were log transformed. Samples are on the x-axis (*A* = *Arillastrum*, *Cor* = *Corymbia*, *H* = *Heteropyxis*, *Mel* = *Melaleuca*), loci are on the y-axis (*chl* = *chloroplast* loci).

Capture sensitivity is defined as the percentage of the target loci that is covered by at least one read [14]. The overall average coverage per sample ranged from 2.8 to 491.6, with an ensemble average of 140. The capture sensitivity of 47 samples across 209 loci was 99.51% (S1 Table). Only 0.49% of the sample-by-locus matrix had zero coverage. By-genus capture sensitivity was: *Eucalyptus* 99.92%, *Melaleuca* 98.80%, *Corymbia* 97.37%, *Arillastrum* 100% and *Heteropyxis* 97.61% (S1 Table). A heat map showing the read coverage across all samples and loci is shown in Fig 4. A total of 83.8% of the data matrix had an average read coverage of at least 20. The average read coverage for chloroplast loci (196.8 reads) was 1.5 times higher than for the nuclear loci (129.5 reads; Table 1). The average read coverage across the 47 samples of chloroplast target loci varied more (3.4–1209.8 reads) than across the nuclear loci (2.1–449.1 reads). The phylogenetic out-group used here, *Heteropyxis*, showed the highest discrepancy between chloroplast (60.03 reads per position) and nuclear (7.48 reads per position) loci coverage.

The enrichment factor for target captures is calculated as following: EF = (Reads on Target/Total Number of Reads)/(Target size/Genome size) [61]. Our enrichment factor is therefore: (26,844,104/46,604,348)/(263,164/(605,000,000+160,137)) = 1,325; based on the nuclear genome size of *E*. *grandis* of 605 Mbp [62] and the *E*. *grandis* chloroplast genome size of 160,137 [63].

### Further capture performance assessments

The p-distance of concatenated chloroplast and nuclear data relative to *E*. *globulus* subsp. *pseudoglobulus* ranged from 0.04 in *E*. sp. (ANBG 9404806), *E*. *globulus* subsp. *maidenii* and *E*. *perriniana* to 0.43 in *Heteropyxis natalensis*, with an average of 0.11 (Table 1). The p-distance between *E*. *globulus* subsp. *pseudoglobulus and C*. *calophylla*, *E*. *goniantha* and *E*. *stenostoma* ranged from 0.29 to 0.47, which was likely due to low number of mapped reads and hence poor quality base calls. Therefore, we excluded these samples from further nucleotide diversity analyses. Another outlier was *H*. *natalensis* (p-distance = 0.43), which is an out-group from the rest of the samples in Myrtaceae according to a previous phylogenetic study [26]. *Heteropixis natalensis* was not excluded because the low coverage in the nuclear targets might have been due to phylogenetic distance rather than due to low number of mapped reads, and it was needed to root the phylogenetic tree.

**Fig 5:**
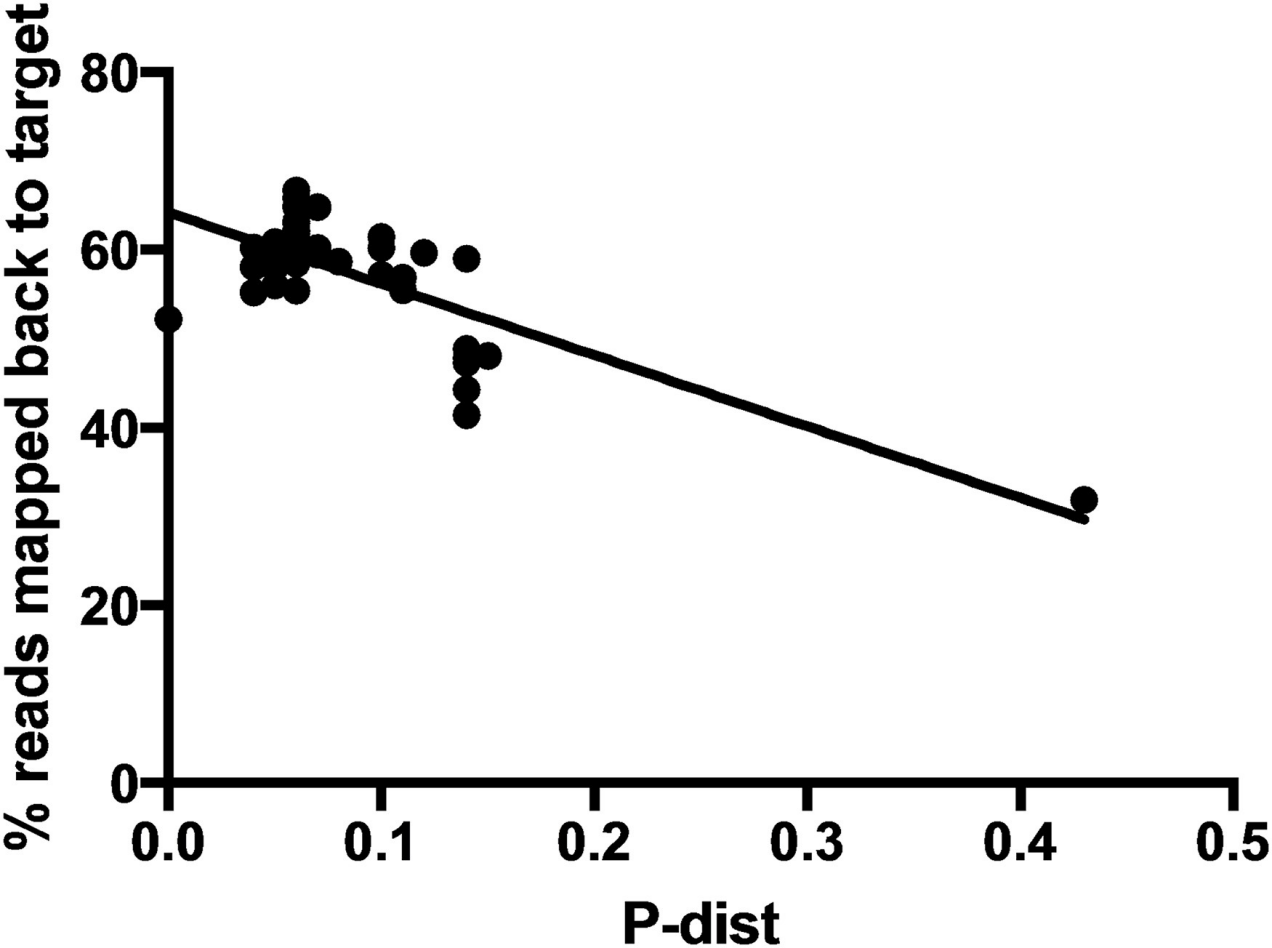
Linear regression of p-distance of each sample from *Eucalyptus globulus* subsp. *pseudoglobulus* versus percentage of reads mapped back to *Eucalyptus grandis* targets. The percentage of reads mapped and the p-distance is significantly negatively correlated (*R*^2^ = 0.36, *P*<0.0001).

Linear regression was used to test whether there is correlation between average coverage and p-distance. Prior to the exclusion of the samples with low number of mapped reads, p-distance and the average coverage had a weak but significant negative correlation (*R*^2^ = 0.158, *P* = 0.0057; data not shown). After removal of those three samples, the negative correlation was stronger (*R*^2^ = 0.36, *P* < 0.0001; Fig 5).

**Fig 6:**
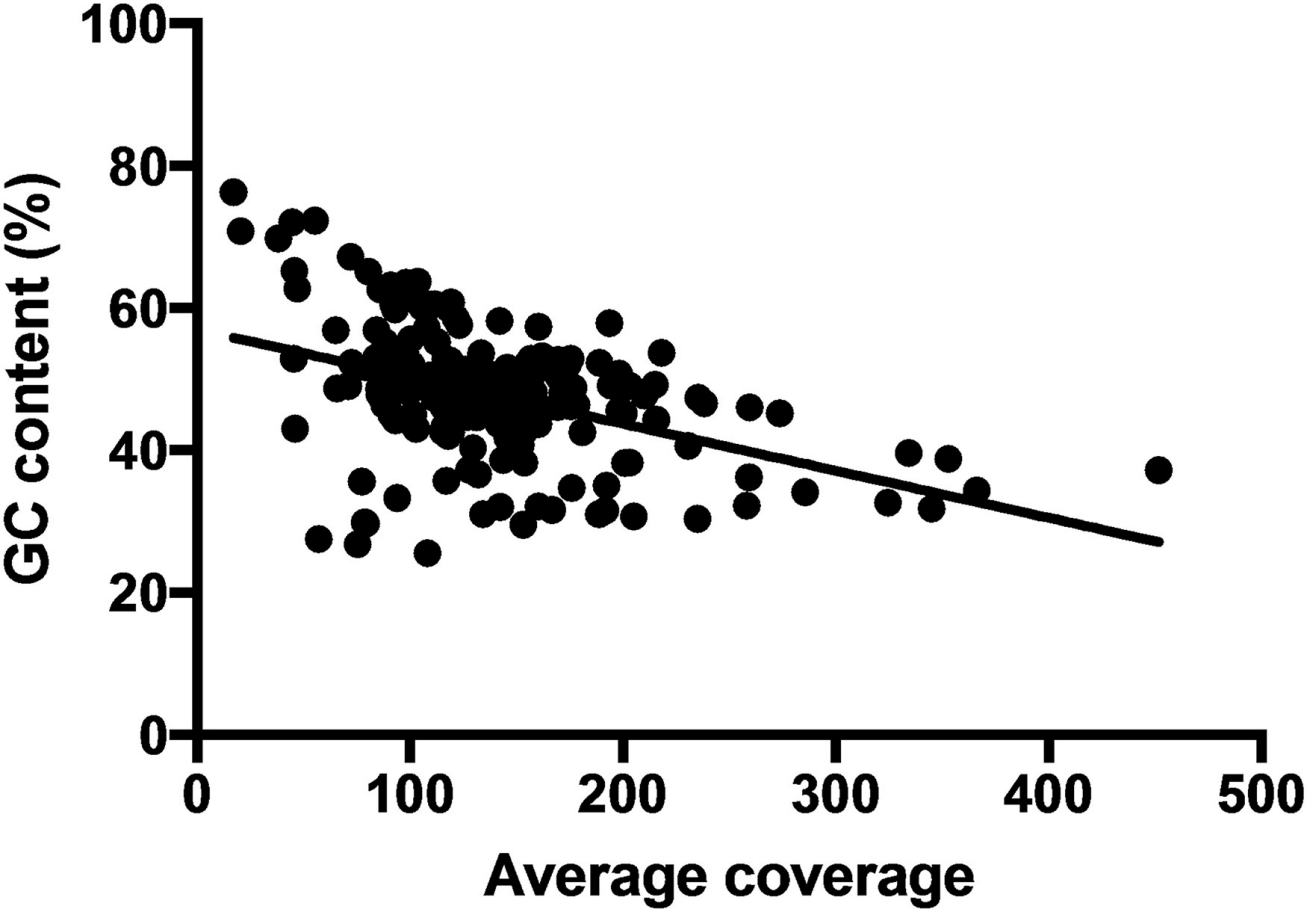
Linear regression of average read coverage versus GC content. GC content and the average coverage are significantly negatively correlated (*R*^2^ = 0.2070, *P* < 0.0001).

We tested whether GC content or locus length had an effect on the average read coverage per locus. A negative correlation was found between GC content and average read coverage (*R*^2^ = 0.2072, *P*<0.0001, Fig 6), but no effect was found for locus length (data not shown).

### Phylogenetic utility

**Fig 7:**
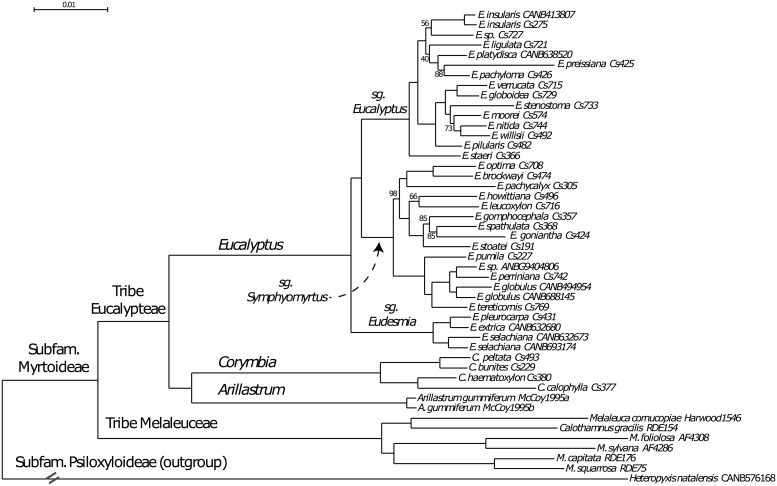
The single maximum likelihood tree of 47 taxa found by multiple searches (see Methods). Bootstrap values are shown and branch labels indicate higher taxonomic groups down to the rank of subgenera (sg). When bootstrap values are not shown, nodes had a bootstrap value of 100.

The number of informative sites of each locus was calculated as a measure of phylogenetic utility (S2 Table). The average number of informative sites in the combined chloroplast and nuclear target regions was 207 per locus. In the chloroplast loci, the number of informative sites ranged from 12 to 193, with an average of 50.7 (7.8%). In the nuclear loci, the number of informative sites varied from 22 to 859, with an average of 236.3 (17.4%) (S2 Table). Four regions (*mat*K-*trn*K, *ndh*F, *psb*A-*trn*H and *trn*L-*trn*F) that had previously been used in phylogenetic studies [31, 64] were found to contain 128.3 informative sites on average (9.9%), slightly more than the chloroplast average. A maximum likelihood tree of the 47 taxa based on 50 independent tree searches using all 209 loci is shown in Fig 7.

## Discussion

In this study, we identified 212 low-copy and orthologous nuclear and chloroplast loci for phylogenetic studies of *Eucalyptus* and *Melaleuca s*.*l*. Sequencing results demonstrate that the target loci were successfully captured in both genera, as well as in three other genera of Myrtaceae (*Arillastrum*, *Corymbia*, *Heteropyxis*). Performance based on specificity and sensitivity across 209 loci was similar to, or better than, that in many other published target capture studies [14, 16]. Exon capture was successful for the majority of samples, with 83.5% of the data matrix having an average coverage of more than 20 for the nuclear loci. We observed a drop down of capture performance for nuclear loci in *Heteropyxis*, which is an out-group of *Melaleuca* and *Eucalyptus*, with a divergence from these two genera of ca. 85 (75–93.2) Ma [26]. The average read coverage for nuclear loci in *Heteropyxis* was 7.5, which allows mapping and genotyping, but with relatively low confidence. Read coverage for the chloroplast loci in this genus remained high however, with an average of 60, as expected for the more slowly evolving chloroplast genome.

Probe design took account of chloroplast DNA being much more abundant in green tissue compared to nuclear DNA. The small ratio difference of mapped reads between the 33 chloroplast and 176 nuclear loci shows that this adjustment was successful and is necessary to obtain a balanced read coverage.

Our target locus selection method showed that loci with very high (>70%) or very low (<30%) GC content had poor capture efficiency. This should be taken into account when designing probes for target capture. The reduced capture efficiency of loci with very high GC content was likely due to reduced hybridization because of formation of secondary structures of the probes [65]. Similar results were shown by Bi et al. [16].

For this study, we quantified libraries prior to pooling from agarose gel electrophoresis images and grouped samples into high or low library concentration. This has strongly influenced the number of reads retrieved per sample. Sample quantification could be improved for example by use of the Agilent bioanalyzer or Qubit fluorometer. Pooling protocols could also be more precise to decrease the variation of reads per sample.

We assessed the number and percentage of parsimonious informative sites from each target as a proxy for phylogenetic utility. Based on the number and the percentage of informative sites, we have successfully identified many nuclear and chloroplast loci for multiple taxonomic depths in Myrtaceae. The average percentage of informative sites in chloroplast markers used in previous studies (9.9%) was slightly higher than that of all chloroplast markers used in this study (7.8%). However, they are within the suitable range of parameters found in a theoretical framework [66].

The tree topology of 47 taxa and 6 genera tested with this method shows high replicability and bootstrap support within clades of genera and subgenera. The tree (Fig 7) is highly congruent with recent phylogenetic analyses of the Myrtaceae and eucalypts [26, 34], as well as the most recent classification of *Eucalyptus s*.*l*. (Nicolle, 2019 (http://www.dn.com.au/Classification-Of-The-Eucalypts.html)). This can be seen when taxa from subfamily rank down to subgenus within *Eucalyptus* are mapped on the tree (Fig 7). Moreover, the inclusion of *Calothamnus* within *Melaleuca* agrees with the independent Sanger-sequencing study by [31].

Our study used multiple genomic resources including an annotated genome of *Eucalyptus* as well as transcriptomes and whole-genome shot-gun sequences from *Melaleuca* species to identify over 200 target nuclear and chloroplast loci. We combined aspects of different published workflows to find orthologous, low copy loci by comparing average coverage of reference sequences across two shot-gun sequences, comparing copy number of loci across *E*. *grandis* and other plant genera, and by assessing species trees for each potential locus for species with known relationships. As more and more annotated genomes, transcriptomes and other genomic resources become available across different groups of organisms, the methods outlined in this study can be applied across various taxonomic levels from any kingdom. Further, the methods outlined here are flexible towards the amount of available genomic resources. We tested the use of two species transcriptome sequences for locus identification without finding any differences. Shot-gun sequencing of species of interest is becoming cheaper all the time and the potential application depends on the genome size of the target taxa, however even for genome sizes in the low gigabase range, it is highly feasible to sequence multiple taxa at low-medium coverage and use for target identification as outlined in this study.

## Supporting information

S1 Table Targeted loci by sample matrix including average read coverage per position and locus information. (XLSX)

S2 Table Information for the targeted loci. (XLSX)

S1 Protocol Detailed target capture protocol. (PDF)

## References

[1] Dong W, Liu J, Yu J, Wang L, Zhou S. Highly Variable Chloroplast Markers for Evaluating Plant Phylogeny at Low Taxonomic Levels and for DNA Barcoding. PLoS ONE. 2012;7(4):e35071 10.1371/journal.pone.0035071 22511980 PMC3325284

[2] Gielly L, Taberlet P. The use of chloroplast DNA to resolve plant phylogenies: noncoding versus rbcL sequences. Molecular Biology and Evolution. 1994;11(5):769–77. 10.1093/oxfordjournals.molbev.a040157 7968490

[3] Akhani H, Malekhammadi M, Gharibiyan A, Chase MW. Phylogenetics of the Irano-Turanian taxa of *Limonium* (Plumbaginaceae) based on ITS nrDNA sequences and leaf anatomy provides evidence for species delimination and relationships of lineages. Botanical Journal of the Linnean Society. 2013;171:519–50.

[4] Zhu W-D, Nie Z-L, Wen J, Sun H. Molecular phylogeny and biogeography of Astilbe (Saxifragaceae) in Asia and eastern North America. Botanical Journal of the Linnean Society. 2013;171(2):377–94. 10.1111/j.1095-8339.2012.01318.x

[5] Townsend TM, Alegre RE, Kelley ST, Wiens JJ, Reeder TW. Rapid development of multiple nuclear loci for phylogenetic analysis using genomic resources: An example from squamate reptiles. Molecular Phylogenetics and Evolution. 2008;47(1):129–42. 10.1016/j.ympev.2008.01.008 18313331

[6] Smith DR. Mutation Rates in Plastid Genomes: They Are Lower than You Might Think. Genome Biology and Evolution. 2015;7(5):1227–34. 10.1093/gbe/evv069 25869380 PMC4453064

[7] Small RL, Cronn RC, Wendel JF. Use of nuclear genes for phylogeny reconstruction in plants. Australian Systematic Botany. 2004;17(2):145–70. 10.1071/SB03015.

[8] Xiang Q-P, Wei R, Shao Y-Z, Yang Z-Y, Wang X-Q, Zhang X-C. Phylogenetic relationships, possible ancient hybridization, and biogeographic history of Abies (Pinaceae) based on data from nuclear, plastid, and mitochondrial genomes. Molecular Phylogenetics and Evolution. 2015;82(Part A):1–14. 10.1016/j.ympev.2014.10.008. 25462996

[9] Boekhorst J, Snel B. Identification of homologs in insignificant blast hits by exploiting extrinsic gene properties. BMC Bioinformatics. 2007;8(1):356 10.1186/1471-2105-8-356 17888146 PMC2048517

[10] Tekaia F. Inferring Orthologs: Open Questions and Perspectives. Genomics Insights. 2016;9:GEI.S37925. 10.4137/gei.s37925 26966373 PMC4778853

[11] Sang T. Utility of Low-Copy Nuclear Gene Sequences in Plant Phylogenetics. Critical Reviews in Biochemistry and Molecular Biology. 2002;37(3):121–47. 10.1080/10409230290771474 12139440

[12] Bragg JG, Potter S, Bi K, Moritz C. Exon capture phylogenomics: efficacy across scales of divergence. Molecular Ecology Resources. 2016;16(5):1059–68. 10.1111/1755-0998.12449 26215687

[13] Rubin BER, Ree RH, Moreau CS. Inferring Phylogenies from RAD Sequence Data. PLoS ONE. 2012;7(4):e33394 10.1371/journal.pone.0033394 22493668 PMC3320897

[14] Peñalba JV, Smith LL, Tonione MA, Sass C, Hykin SM, Skipwith PL, et al Sequence capture using PCR-generated probes: a cost-effective method of targeted high-throughput sequencing for nonmodel organisms. Molecular Ecology Resources. 2014;14(5):1000–10. 10.1111/1755-0998.12249 24618181

[15] Tonnabel J, Olivieri I, Mignot A, Rebelo A, Justy F, Santoni S, et al Developing nuclear DNA phylogenetic markers in the angiosperm genus Leucadendron (Proteaceae): A next-generation sequencing transcriptomic approach. Molecular Phylogenetics and Evolution. 2014;70:37–46. 10.1016/j.ympev.2013.07.027 23948865

[16] Bi K, Vanderpool D, Singhal S, Linderoth T, Moritz C, Good JM. Transcriptome-based exon capture enables highly cost-effective comparative genomic data collection at moderate evolutionary scales. BMC Genomics. 2012;13(1):403 10.1186/1471-2164-13-403 22900609 PMC3472323

[17] Li C, Ortí G, Zhang G, Lu G. A practical approach to phylogenomics: the phylogeny of ray-finned fish (Actinopterygii) as a case study. BMC Evolutionary Biology. 2007;7(1):44 10.1186/1471-2148-7-44 17374158 PMC1838417

[18] Portik DM, Smith LL, Bi K. An evaluation of transcriptome-based exon capture for frog phylogenomics across multiple scales of divergence (Class: Amphibia, Order: Anura). Molecular Ecology Resources. 2016;16(5):1069–83. 10.1111/1755-0998.12541 27241806

[19] Lemmon EM, Lemmon AR. High-Throughput Genomic Data in Systematics and Phylogenetics. Annual Review of Ecology, Evolution, and Systematics. 2013;44(1):99–121. 10.1146/annurev-ecolsys-110512-135822

[20] Faircloth BC, McCormack JE, Crawford NG, Harvey MG, Brumfield RT, Glenn TC. Ultraconserved Elements Anchor Thousands of Genetic Markers Spanning Multiple Evolutionary Timescales. Systematic biology. 2012;61(5):717–26. 10.1093/sysbio/sys004 22232343

[21] Lemmon AR, Lemmon EM. High-Throughput Identification of Informative Nuclear Loci for Shallow-Scale Phylogenetics and Phylogeography. Systematic biology. 2012;61(5):745–61. 10.1093/sysbio/sys051 22610088

[22] Weitemier K, Straub SCK, Cronn RC, Fishbein M, Schmickl R, McDonnell A, et al Hyb‐Seq: Combining target enrichment and genome skimming for plant phylogenomics. Applications in Plant Sciences. 2014;2(9):1400042 10.3732/apps.1400042 25225629 PMC4162667

[23] Schmickl R, Liston A, Zeisek V, Oberlander K, Weitemier K, Straub SCK, et al Phylogenetic marker development for target enrichment from transcriptome and genome skim data: the pipeline and its application in southern African Oxalis (Oxalidaceae). Molecular Ecology Resources. 2016;16(5):1124–35. 10.1111/1755-0998.12487 26577756

[24] Kadlec M, Bellstedt DU, Le Maitre NC, Pirie MD. Targeted NGS for species level phylogenomics: “made to measure” or “one size fits all”? PeerJ. 2017;5:e3569 10.7717/peerj.3569 28761782 PMC5530999

[25] Thornhill AH, Crisp MD. Phylogenetic assessment of pollen characters in Myrtaceae Australian Systematic Botany. 2012;25:171–87.

[26] Thornhill AH, Ho SYW, Külheim C, Crisp MD. Interpreting the modern distribution of Myrtaceae using a dated molecular phylogeny. Molecular Phylogenetics and Evolution. 2015;93:29–43. 10.1016/j.ympev.2015.07.007 26211451

[27] Steane DA, Nicolle D, McKinnon GE, Vaillancourt RE, Potts BM. Higher-level relationships among the eucalypts are resolved by ITS-sequence data. Australian Systematic Botany. 2002;15(1):49–62. 10.1071/SB00039.

[28] Lucas EJ, Harris SA, Mazine FF, Belsham SR, Lughadha EMN, Telford A, et al Suprageneric Phylogenetics of Myrteae, the Generically Richest Tribe in Myrtaceae (Myrtales). Taxon. 2007;56(4):1105–28. 10.2307/25065906

[29] Cook LG, Morris DC, Edwards RD, Crisp MD. Reticulate evolution in the natural range of the invasive wetland tree species Melaleuca quinquenervia. Molecular Phylogenetics and Evolution. 2008;47(2):506–22. 10.1016/j.ympev.2008.02.012 18387315

[30] Biffin E, Harrington MG, Crisp MD, Craven LA, Gadek PA. Structural partitioning, paired-sites models and evolution of the ITS transcript in Syzygium and Myrtaceae. Molecular Phylogenetics and Evolution. 2007;43(1):124–39. 10.1016/j.ympev.2006.08.013 17070713

[31] Edwards RD, Craven LA, Crisp MD, Cook LG. Melaleuca revisited: cpDNA and morphological data confirm that Melaleuca L. (Myrtaceae) is not monophyletic. Taxon. 2010;59(3):744–54.

[32] Crisp MD, Burrows GE, Cook LG, Thornhill AH, B D.M. Flammable biomes dominated by eucalypts originated at the Cretaceous-Palaeogene boundary. Nature Comm. 2012;2:193. 10.1038/ncomms1191 21326225

[33] Bayly MJ, Rigault P, Spokevicius A, Ladiges PY, Ades PK, Anderson C, et al Chloroplast genome analysis of Australian eucalypts–Eucalyptus, Corymbia, Angophora, Allosyncarpia and Stockwellia (Myrtaceae). Molecular Phylogenetics and Evolution. 2013;69(3):704–16. 10.1016/j.ympev.2013.07.006 23876290

[34] Thornhill AH, Crisp MD, Külheim C, Lam KE, Nelson LA, Yeates DK, et al A dated molecular perspective of eucalypt taxonomy, evolution and diversification. Australian Systematic Botany. 2019;32(1):29–48. 10.1071/SB18015.

[35] Schuster TM, Setaro SD, Tibbits JFG, Batty EL, Fowler RM, McLay TGB, et al Chloroplast variation is incongruent with classification of the Australian bloodwood eucalypts (genus Corymbia, family Myrtaceae). PLOS ONE. 2018;13(4):e0195034 10.1371/journal.pone.0195034 29668710 PMC5905893

[36] Hsieh J-F, Chuah A, Patel HR, Sandhu K, Foley WJ, Külheim C. Transcriptome Profiling of Melaleuca quinquenervia Challenged by Myrtle Rust Reveals Differences in Defense Responses among Resistant Individuals. Phytopathology. 2017 10.1094/phyto-09-17-0307-r 29135360

[37] Grabherr MG, Haas BJ, Yassour M, Levin JZ, Thompson DA, Amit I, et al Full-length transcriptome assembly from RNA-Seq data without a reference genome. Nat Biotech. 2011;29(7):644–52. http://www.nature.com/nbt/journal/v29/n7/abs/nbt.1883.html#supplementary-information. 10.1038/nbt.1883 PMC3571712 21572440

[38] Li W, Godzik A. Cd-hit: a fast program for clustering and comparing large sets of protein or nucleotide sequences. Bioinformatics. 2006;22(13):1658–9. 10.1093/bioinformatics/btl158 16731699

[39] Doyle JJ, Dickson EE. Preservation of Plant Samples for DNA Restriction Endonuclease Analysis. Taxon. 1987;36(4):715–22. 10.2307/1221122

[40] Grattapaglia D, Vaillancourt R, Shepherd M, Thumma BR, Foley WJ, Külheim C, et al Progress in Myrtaceae genetics and genomics: Eucalyptus as the pivotal genus. Tree Genetics & Genomes. 2012;8(3):463–508. 10.1007/s11295-012-0491-x

[41] Goodstein DM, Shu S, Howson R, Neupane R, Hayes RD, Fazo J, et al Phytozome: a comparative platform for green plant genomics. Nucleic Acids Research. 2012;40(D1):D1178–D86. 10.1093/nar/gkr944 22110026 PMC3245001

[42] Kearse M, Moir R, Wilson A, Stones-Havas S, Cheung M, Sturrock S, et al Geneious Basic: An integrated and extendable desktop software platform for the organization and analysis of sequence data. Bioinformatics. 2012;28(12):1647–9. 10.1093/bioinformatics/bts199 22543367 PMC3371832

[43] Craven LA, Edwards RD, Cowley KJ. New combinations and names in Melaleuca (Myrtaceae). Taxon. 2014;63(3):663–70. 10.12705/633.38

[44] Rohland N, Reich D. Cost-effective, high-throughput DNA sequencing libraries for multiplexed target capture. Genome Research. 2012;22(5):939–46. 10.1101/gr.128124.111 22267522 PMC3337438

[45] Dodt M, Roehr J, Ahmed R, Dieterich C. FLEXBAR—Flexible Barcode and Adapter Processing for Next-Generation Sequencing Platforms. Biology. 2012;1(3):895 10.3390/biology1030895 24832523 PMC4009805

[46] Katoh K, Standley DM. MAFFT Multiple Sequence Alignment Software Version 7: Improvements in Performance and Usability. Molecular Biology and Evolution. 2013;30(4):772–80. 10.1093/molbev/mst010 23329690 PMC3603318

[47] Tamura K, Stecher G, Peterson D, Filipski A, Kumar S. MEGA6: Molecular Evolutionary Genetics Analysis Version 6.0. Molecular Biology and Evolution. 2013;30(12):2725–9. 10.1093/molbev/mst197 24132122 PMC3840312

[48] Misof B, Misof K. A Monte Carlo approach successfully identifies randomness in multiple sequence alignments: a more objective means of data exclusion. Systematic biology. 2009;58(1):21–34. Epub 2009/02/01. 10.1093/sysbio/syp006 . 20525566

[49] Kück P, Meusemann K, Dambach J, Thormann B, von Reumont BM, Wägele JW, et al Parametric and non-parametric masking of randomness in sequence alignments can be improved and leads to better resolved trees. Frontiers in Zoology. 2010;7(1):10 10.1186/1742-9994-7-10 20356385 PMC2867768

[50] Ho SY, Jermiin L. Tracing the decay of the historical signal in biological sequence data. Systematic biology. 2004;53(4):623–37. Epub 2004/09/17. 10.1080/10635150490503035 . 15371250

[51] Jermiin L, Ho SY, Ababneh F, Robinson J, Larkum AW. The biasing effect of compositional heterogeneity on phylogenetic estimates may be underestimated. Systematic biology. 2004;53(4):638–43. Epub 2004/09/17. 10.1080/10635150490468648 . 15371251

[52] Lanfear R, Frandsen PB, Wright AM, Senfeld T, Calcott B. PartitionFinder 2: New Methods for Selecting Partitioned Models of Evolution for Molecular and Morphological Phylogenetic Analyses. Mol Biol Evol. 2017;34(3):772–3. Epub 2016/12/26. 10.1093/molbev/msw260 . 28013191

[53] Stamatakis A. RAxML version 8: a tool for phylogenetic analysis and post-analysis of large phylogenies. Bioinformatics. 2014;30(9):1312–3. Epub 2014/01/24. 10.1093/bioinformatics/btu033 . 24451623 PMC3998144

[54] Hurvich C, Tsai C. Regression and time-series model selection in small samples. Biometrika. 1989;76:297–307.

[55] Kalyaanamoorthy S, Minh BQ, Wong TKF, von Haeseler A, Jermiin LS. ModelFinder: fast model selection for accurate phylogenetic estimates. Nat Methods. 2017;14(6):587–9. Epub 2017/05/10. 10.1038/nmeth.4285 . 28481363 PMC5453245

[56] Nguyen LT, Schmidt HA, von Haeseler A, Minh BQ. IQ-TREE: a fast and effective stochastic algorithm for estimating maximum-likelihood phylogenies. Mol Biol Evol. 2015;32(1):268–74. Epub 2014/11/06. 10.1093/molbev/msu300 . 25371430 PMC4271533

[57] Chernomor O, von Haeseler A, Minh BQ. Terrace Aware Data Structure for Phylogenomic Inference from Supermatrices. Systematic biology. 2016;65(6):997–1008. Epub 2016/04/29. 10.1093/sysbio/syw037 . 27121966 PMC5066062

[58] Pattengale ND, Alipour M, Bininda-Emonds OR, Moret BM, Stamatakis A. How many bootstrap replicates are necessary? J Comput Biol. 2010;17(3):337–54. Epub 2010/04/10. 10.1089/cmb.2009.0179 . 20377449

[59] Aberer AJ, Krompass D, Stamatakis A. Pruning rogue taxa improves phylogenetic accuracy: an efficient algorithm and webservice. Systematic biology. 2013;62(1):162–6. Epub 2012/09/11. 10.1093/sysbio/sys078 . 22962004 PMC3526802

[60] Gouy M, Guindon S, Gascuel O. SeaView version 4: A multiplatform graphical user interface for sequence alignment and phylogenetic tree building. Mol Biol Evol. 2010;27(2):221–4. Epub 2009/10/27. 10.1093/molbev/msp259 . 19854763

[61] Mertes F, ElSharawy A, Sauer S, van Helvoort JMLM, van der Zaag PJ, Franke A, et al Targeted enrichment of genomic DNA regions for next-generation sequencing. Briefings in Functional Genomics. 2011;10(6):374–86. 10.1093/bfgp/elr033 22121152 PMC3245553

[62] Myburg AA, Grattapaglia D, Tuskan GA, Hellsten U, Hayes RD, Grimwood J, et al Genome sequence of Eucalyptus grandis: A global tree crop for fiber and energy. Nature. 2013.

[63] Paiva JA, Prat E, Vautrin S, Santos MD, San-Clemente H, Brommonschenkel S, et al Advancing Eucalyptus genomics: identification and sequencing of lignin biosynthesis genes from deep-coverage BAC libraries. BMC Genomics. 2011;12(1):137 10.1186/1471-2164-12-137 21375742 PMC3060884

[64] Wilson PG, O’Brien MM, Gadek PA, Quinn CJ. Myrtaceae Revisited: A Reassessment of Infrafamilial Groups. American Journal of Botany. 2001;88(11):2013–25. 10.2307/3558428 21669634

[65] Mamanova L, Coffey AJ, Scott CE, Kozarewa I, Turner EH, Kumar A, et al Target-enrichment strategies for next-generation sequencing. Nature Methods. 2010;7:111 10.1038/nmeth.1419 https://www.nature.com/articles/nmeth.1419#supplementary-information. 20111037

[66] Klopfstein S, Massingham T, Goldman N. More on the Best Evolutionary Rate for Phylogenetic Analysis. Systematic biology. 2017;66(5):769–85. 10.1093/sysbio/syx051 28595363 PMC5790136

