# Supplementary material for: Identifying genetic markers for a range of phylogenetic utility–From species to family level
Source: PLoS One. 2019 Aug 1;14(8):e0218995. doi: 10.1371/journal.pone.0218995 (PMC6675087; doi:10.1371/journal.pone.0218995)
Supplement: S1 Protocol — (PDF) [file pone.0218995.s003.pdf]

Supplementary laboratory protocol for exon capture

The overall work flow (sera mag clean up after each step) followed Rohland and Reich (2012), but the details of each step (except for) sera mag clean up after each reaction followed Maricic (2010). Modifications are highlighted in Bold.

Summary of workflow

1. Shearing
2. 1.6X Sera-Mag Bead clean up
3. Blunt end repair
4. 2X Sera-Mag Bead clean up
5. Adapter ligation
6. 1.6X Sera-Mag Bead clean up
7. Nick fill in
8. 1.6X Sera-Mag Bead clean up
9. Enrichment PCR
10. Gel electrophoresis and pooling
11. 1.2X Sera-Mag Bead clean up
12. Hybridisation
13. Recovery and wash
14. Semi-qPCR
15. Indexing PCR
16. 1.2X Sera-Mag Bead clean up
17. Gel run and Qubit quantification
18. Final pooling of indexed library

Reagents

Agarose and agarose gel reagents
Bst large fragment polymerase
DNA ladder (100bp, e.g. Axygen cat.no. M-DNA-100bp)
Dynabeads M-270 Streptavidin (Invitrogen cat.no. 65305)
Ethanol 100%
Orange loading dye
HotStarTaq Plus DNA polymerase (Qiagen cat.no. 203603)
Human COT-1 DNA (Invitrogen, cat.no. 15279-011)
Milli Q water
Phusion Hot Start II DNA polymerase (Thermo Scientific cat.no. F-549S)
Qubit dsDNA HS Assay Kit (Invitrogen cat.no. Q32854)
Quick Blunting Kit (New England BioLabs cat.no. E1201L)
Quick Ligatioin Kit (New England BioLabs cat.no. M2200L)
SeqCap EZ Developer Library (NimbleGen, 06471714001)
SeqCap Developer Reagent (NimbleGen, 06684335001)
SeqCap Hybridisation and Wash Kit (NimbleGen, 05634261001)
Sera-Mag SpeedBeads carboxylate-modified particles (GE Healthcare Life cat.no. 65152105050250)
Tris (10mM, pH 8.5)

Equipment

Agarose gel electrophoresis unit
DNA shearing device (e.g. Bioruptor NGS, Diagenode)

PCR plates (96-Well, 200 μL) and seals
Filter tips
Ice
LabChip GX II (Caliper Life Sciences)
LabChip DS system (Caliper Life Sciences)
Ring magnet stand (e.g. SPRIplate 96-Ring, Agencourt)
PCR machines
PCR 8-tube strips and caps (e.g. Axygen cat. no. PCR-02-FCP-C, PCR-0208-C)
Pipettor (single-channel, multi-channel)
Qubit 2.0 Fluorometer (Invitrogen, 1011007409)
Speed vacuum concentrator
Tube (Bioruptor 0.65mL mitrotubes for DNA shearing cat.no. C30010011)
Tube (microcentrifuge, 1.5 mL e.g. Eppendorf)
Tube (Qubit assay, Invitrogen Q32856, 500 μL)
Vortex mixer

Reagent preparations
Preparation of Sera-Mag Bead (See Faircloth and Glenn 2014)
10X Blunting buffer (See Rohland and Reich 2012, 100 mM $MgCl_2$, 500 mM Tris-HCl pH 7.5, 75 mM DTT, dNTPs)
Preparation of barcoded-P5-adapter
Preparation PE-P7-adapter

Note
For the optimal results, it is suggested that the whole procedures are done consecutively by minimizing the time samples being stored in the freezer.
The library preparation was done on a PCR plate and the TIMING is per plate (96 samples) based.

Workflow

1. Shearing of Diluted DNA TIMING 8h
Shear 100 μL of 6 ng/μL of diluted genomic DNA using Bioruptor ultrasonicator using 30 cycles of 30 sec on and off with intensity set to H(igh).

2. 1.6X Sera-Mag Bead Clean Up TIMING 2h (from manufacturer's protocol of Agencourt AMPure XP and also reference Rohland and Reich 2012)
a) Pull the Sera-Mag Beads out 20 min in advance so that the beads are at room temperature. Vortex the beads before use.

b) Add 1.6X volumes (160 μL) of Sera-Mag Beads to each well of PCR plate using a multi-channel pipettor. Pipette mix 10X.

c) Incubate the sample at room temperature for 10 min in dark.

d) Put the PCR plate on the ring magnet stand and wait until the liquid goes clear.

e) Remove and discard the supernatant.

f) Remove the magnetic plate.

e) Add 200 µL of fresh 80% ethanol and leave in for 1 min on a magnetic plate. Remove and discard the supernatant.

f) Repeat the ethanol wash in total of two washes.

g) Incubate the samples in a fume hood for 10-15 min uncovered until the pellet is dry.

h) Add 24.63 µL of 10mM Tris (pH 8.5) and resuspend by pipette mixing 10X.

i) Incubate at room temperature for 5 min. Spin down.

j) Put the PCR plate on the ring magnet stand and incubate at room temperature for 5 min.

k) Transfer 23.63 µL of the cleaned up DNA to a new PCR plate.

3. Blunt End Repair TIMING 1.5h (from Rohland and Reich 2012)
a) Prepare the mix as below. Perform the following reaction in a 96-well PCR plate.

Reagent	Volume (µL) per sample	Final concentration
Sheared DNA (0.6µg)	23.63	
dNTPs (25mM)	0.12	0.1mM
**ATPs (10mM)**	3	1mM
Blunting buffer (10X)	3	1X
Blunting enzyme mix	0.25	
Total volume	30 µL	

b) Incubate for 20 min at 12°C, followed by 15 min at 37°C.

4. 2X Sera-Mag Bead Clean Up TIMING 2h
Perform the same as the first Sera-Mag Bead clean up except initially adding 2X (60 µL) Sera-Mag Beads and using 70% EtOH for a total of two washes. Elute in 11.66 µL MiliQ water, and transfer 10.66 µL at the final step.

5. Adapter Ligation TIMING 1.5h (from Rohland and Reich 2012)
a) Create the following mix. Perform the following reaction in a 96-well PCR plate.

Reagent	Volume (µL) per sample	Final concentration
Blunted DNA	20.34	
Quick Ligase Buffer (2X)	15	0.75X
Quick T4 DNA Ligase	**1**	
Barcoded-P5-adapter	1.67	
PE-P7-adapter	1.67	
Total volume	30 µL	

b) Incubate for 30 min at 22-24°C.

6. 2X Sera-Mag Bead Clean Up TIMING 2h
Perform the same as the first Sera-Mag Bead clean up except initially adding 2X (60 μL) Sera-Mag Beads and use 70% EtOH for the two times washes. Elute in 27.63 μL MiliQ water, and transfer 26.63 μL at the final step.

7. Nick Fill-in TIMING 1.5h (from Rohland and Reich 2012)
a) Prepare the master mix as below. Perform the following reaction in a 96-well plate.

Reagent	Volume (μL) per sample	Final concentration
Ligated DNA	26.63	
dNTPs (25mM)	0.3	4mM
ThermoPol Reaction Buffer (10X)	3	1X
Bst Polymerase large fragment (8000U/mL)	0.067	240U
Total volume	30 μL	

b) Incubate for **20 min** at 37°C.

8. 1.6X Sera-Mag Bead Clean Up TIMING 2h
Perform the same as the first Sera-Mag Bead clean up except initially adding 1.6X (48 μL) Sera-Mag Beads and using 70% EtOH for a total of two washes. Elute in 17.51 μL MiliQ water, and transfer 16.51 μL at the final step.

9. Enrichment PCR TIMING 1.5h

a) Prepare the reaction mix as indicated below and perform the reaction in a 96-well PCR plate.

Reagent	Volume (μL) per sample	Final concentration
Nick filled-in DNA	16.51	
PCR buffer (10X)	2	1X
$MgCl_2$ (25mM)	0.8	1mM
PreHyb-PE_F&R (24μM)	0.2	0.25 μM
dNTP (25mM)	0.16	0.2mM
HotStarTaq Plus DNA Polymerase (5U/μL)	0.33	0.08U/μL
Total volume	20 μL	

b) Run the program as below in a thermocylcler.
6 min 95°C
(20 sec 95°C, 45 sec 55°C, 30 sec 72°C) **X 13***
5 min 72°C
∞ 4°C
*The number of cycles can be adjusted to 10-15 depending on the concentration of the DNA.

10. Gel Electrophoresis and Pooling TIMING 4h

a) Prepare 1.2% agarose gel + EtBr. Load 3µL of PCR product with orange loading dye and 2.5µL of 100bp DNA ladder on the gel.

b) Pool the following volume of *48 samples according to the band intensity of the gel eletrophoresis image.

Strong: 3-5 µL (DNA is visible as a dark band)

Medium: 9-10 µL (DNA visible as a band but not as intense as Strong bands)

Weak: 13-16 µL (DNA invisible)

*We worked on 48 samples per a pool. The number of samples per pool can be adjusted.

11. Sera-Mag Bead Clean up (1.2X) TIMING 2h
Perform the same as the first Sera-Mag Bead clean up except initially adding 1.2X Sera-Mag Beads of enrichment PCR products and using 70% EtOH for a total of two washes. Elute in 51 µL MiliQ water to transfer 50 µL at the final step.

12. Hybridisation TIMING 76.5h (Roche NimbleGen, Chapter 5. SeqCap EZ Library SR User's Guide)
a) Prepare the hybridization mix as below.

Reagent	Volume (µL) per sample	Final concentration
Pooled and cleaned up DNA library	50	
**BO1.P5.F (200µM)**	0.83	2.4µM
**BO2.P5.R (200µM)**	0.83	2.4µM
**BO3.P7.part1.F (200µM)**	0.83	2.4µM
**BO4.P7.part1.R (200µM)**	0.83	2.4µM
**BO5.P7.part2.F (200µM)**	0.83	2.4µM
**BO6.P7.part2.R (200µM)**	0.83	2.4µM
**Univ_Block_P5 (200**µM)	2.5	7.2µM
**Univ_Block_P7 (200**µM)	2.5	7.2µM
**Human COT-1 DNA (1mg/mL)**	5	0.07mg/mL
**Nimblegen Seq Capture EZ developer reagent**	5	
Total volume	70 µL	

b) Add 20 µL of hybridisation mix to 49 µL of pooled and cleaned up DNA library in a PCR tube.

c) **Make holes on the tube using a needle.**

d) **Speed vac until dry.**

e) Change the lid so that the tube does not have a hole.

f) **Add the following reagents to the pooled and cleaned up DNA and hybridization mix.**

Reagent	Volume (μL) per sample
Hybridisation buffer (2X) (vial5)	7.5
Hybridisation component A (vial6)	3
Ez developer library	4.5
Total volume	15 μL

g) **Mix well to dissolve.**

h) **Dissolve the sample at 95°C on a thermocycler up to 45 min. Mix by pipetting every 15 min.**

i) **After the sample is dissolved, incubate at 95°C for 10 min.**

j) **Start gradual temperature drop down from 95°C to 52°C (-0.1°C/sec).**

k) Once the temperature reaches 52°C quickly set the temperature to 47°C and leave it for incubation for 72hrs.

13. Wash and Recovery of Captured Sample TIMING 2.5h (NimbleGen, Chapter 6. SeqCap EZ Library SR User's Guide)
a) Pull out the streptavidin beads 20 min before use.

b) Prepare 1X working buffers of each concentrated wash buffer as directed below. Prepare extra 5% volume of the 1X working buffers as suggested below.

Concentrated buffer	Buffer (μL)	MiliQ water (μL)	Total volume
10X wash buffer I (vial 1)	31.5	283.5	315 μL
10X wash buffer II (vial 2)	21	189	210 μL
10X wash buffer III (vial 3)	21	189	210 μL
10X stringent wash buffer (vial 4)	42	378	420 μL
2.5X bead wash buffer (vial 7)	210	315	525 μL

c) Aliquot 400 μL of 1X stringent wash buffer and 100 μL of 1X wash buffer I into each well of a PCR strip tube. **Heat** the two buffers and **magnetic bars** on a pcr machine at 47°C.

STEP 2. Prepare the Capture Beads

d) Mix the streptavidin beads well by vortexing.

e) Aliquot 100 μL of the streptavidin beads into a pcr tube.

f) Place the tube on the ring magnet stand.

g) Remove the supernatant when it goes clear.

h) Add 2X volume (200 μL) of bead wash buffer (vial 7) to beads on magnet.

i) Remove magnet and mix by pipetting.

j) Place the tube back in the magnet.

k) Discard supernatant when clear.

l) Repeat Step h)-k) for a total of two washes.

m) **After removing the supernatant add 100 μL of bead wash buffer (vial 7).**

n) **Incubate at 47°C on a PCR machine.**

o) **Place the heated bar magnet.**

p) Once clear remove the supernatant. Beads are ready.

STEP 3. Bind DNA to the Capture Beads

a) Straight away transfer 15 μL hybridisation sample to the beads on PCR machine at 47°C. Do not let the beads dry.

b) Mix thoroughly ten times by pipetting up and down.

c) Incubate for 45 min at 47°C.

d) Mix by pipetting for 3 sec at 15 min interval.

STEP 4. Wash the Captured Beads Plus Bound DNA

a) After the 45 min incubation, add 100 μL wash buffer I (vial 1) heated to 47°C to the bead plus captured DNA.

b) Mix by pipetting five times.

c) Place a heated bar magnet.

d) Remove supernatant once clear.

e) Remove the magnet.

f) Add 200 µL 1X stringent wash buffer (vial 4) heated to 47°C.

g) Mix by pipetting five times.

h) Incubate at 47°C for 5 min.

i) Place a heated bar magnet.

j) Remove supernatant once clear.

k) Repeat step f)-j) for a total of two washes.

l) Once the supernatant is removed, all the rest of the steps are done at room temperature.

m) Add 200 µL room temp wash buffer I (vial 1).

n) Mix by pipetting a few times then vortex for 2 min. Give PCR tube a quick hand spin.

o) Place the tube in a magnetic plate to bind the beads.

p) Remove and discard supernatant once clear.

q) Add 200 µL of room temperature wash buffer II (vial2).

r) Mix by vortexing for 1 min and do a quick hand spin.

l) Place the tube on the ring magnetic plate.

m) Remove and discard supernatant once clear.

n) Add 200 µL of room temperature buffer III (vial 3)

o) Mix by vortexing for 30 sec.

p) Place the tube on the ring magnetic plate.

q) Remove and discard liquid once clear.

r) Remove the tube from the ring magnetic plate.

s) Add 60 μL 0.1mM EDTA.

t) Store samples captured plus beads0 in a freezer.

14. Semi-qPCR TIMING 3h
a) Prepare the reaction mix.

Reagent	Volume (μL) per sample	Final concentration
Washed and recovered captured library	10	
MiliQ water	4.2	
Phusion HotStart HF buffer (5X)	4	0.4X
$MgCl_2$ (50mM)	0.4	1mM
Sol-PE-PCR_F (10μM)	0.4	0.2 μM
Sol-PE-PCR_R (10μM)	0.4	0.2 μM
Phusion HotStart HF DNA polymerase (2U/μL)	0.4	0.02U/μL
Total volume	49.8	

b) Run the following program (a total of 25 cycles). After 15, 20, 25 cycle remove 4 μL of PCR product while the temperature is at 72°C to check the concentration at each cycle.
1 min 98°C
(15 sec 98°C, 30 sec 60°C, 30 sec 72°C) X 15 cycles
2 min 72°C
(15 sec 98°C, 30 sec 60°C, 30 sec 72°C) X 5 cycles
2 min 72°C
(15 sec 98°C, 30 sec 60°C, 30 sec 72°C) X 5 cycles
7 min 72°C
∞ 4°C

c) Prepare 1.2% Agarose gel + EtBr. Load 4 μL of the PCR product with orange loading dye and 2.5 μL of 100bp DNA ladder to run the gel.

* We estimated the number of indexing PCR at this step. The remaining semi-qPCR product from the 25 cycles can be used for Qubit quantification after Sera-Mag Bead clean up.

15. Indexing PCR TIMING 1.5h
a) Prepare the mix as below.

Reagent	Volume (μL) per sample	Final concentration
Captured DNA libraries	10	
MiliQ water	3.8	
Phusion HotStart HF	4	1X

buffer (5X)		
dNTPs (25mM)	0.2	0.26mM
Sol-PE-PCR_F (10µM)	0.4	0.2 µM
Index-XX-PE Primer (5µM)	0.8	0.2 µM
Phusion HotStart HF DNA polymerase (2U/µL)	0.4	0.04U/ µL
Total volume	19.6 µL	

b) Run the following program:
1 min 98°C
(30 sec 98°C, 15 sec 60°C, 15 sec 72°C) X # cycles decided from semi-qPCR
7min 72°C
∞ 4°C

16. Sera-Mag Bead Clean up (1.2X) TIMING 2h
Perform the same as the first Sera-Mag Bead Clean up except initially adding 1.2X (24 µL) Sera-Mag beads and using 70% EtOH for a total of two washes. Elute in 25 µL of MiliQ water, and transfer 24 µL at the final step.

17. Gel Run and Qubit Quantification TIMING 1.5h
a) Prepare 1.2% agarose gel + EtBr. Load 3 µL of PCR product with orange loading dye and 2.5 µL of 200 bp DNA ladder to run the gel.

b) Quantify the concentration of PCR product (2µL) using Qubit fluorometric quantification following the manufacturer's protocol.

18. Final Pooling and Submission of Indexed Library for Sequencing
Prepare 2 nM of 60 µL of indexed library in MiliQ water.

References

Agencourt AMPure XP Instructions For Use

Faircloth, BC, Glenn, TC. 2014. Protocol: Preparation of an AMPure XP substitute (AKA Serapure). doi: 10.6079/J9MW2F26.

Maricic, T., Whitten, M., Pääbo, S. 2010. Multiplexed DNA Sequence capture of mitochondrial genomes using PCR products. *PLoS One, 5*, e14004

Peñalba, J. V., Smith, L. L., Tonione, M. A., Sass, C., Hykin, S. M., Skipwith, P. L., Moritz, C. 2014. Sequence capture using PCR-generated probes: a cost-effective method of targeted high-throughput sequencing for nonmodel organisms. *Molecular Ecology Resources, 14*(5), 1000-1010.

Rohland and Reich 2012 Cold Spring Harbor protocols

SeqCap EZ Library SR User's Guide
